# Phenolipids, Amphipilic Phenolic Antioxidants with Modified Properties and Their Spectrum of Applications in Development: A Review

**DOI:** 10.3390/biom12121897

**Published:** 2022-12-17

**Authors:** Silvia Ivonne Arzola-Rodríguez, Laila-Nayzzel Muñoz-Castellanos, César López-Camarillo, Erika Salas

**Affiliations:** 1 Facultad de Ciencias Químicas, Universidad Autónoma de Chihuahua, Chihuahua C.P. 31125, Mexico; 2 Laboratorio de Oncogenómica y Proteómica del Cáncer, Universidad Autónoma de la Ciudad de México, Ciudad de México C.P. 03100, Mexico

**Keywords:** polyphenolic compounds, lipophilization, phenolipids, amphipilic molecules, antioxidant capacity, biological activity

## Abstract

Polyphenols, as secondary metabolites from plants, possess a natural antioxidant capacity and biological activities attributed to their chemical and structural characteristics. Due to their mostly polar character, polyphenols present a low solubility in less polar environments or hydrophobic matrices. However, in order to make polyphenols able to incorporate in oils and fats, a transformation strategy is necessary. For the above, the functionalization of polyphenols through chemical or enzymatic lipophilization has allowed the synthesis of phenolipids. These are amphipilic molecules that preserve the natural phenolic core to which an aliphatic motif is attached by esterification or transesterification reactions. The length of the aliphatic chain in phenolipids allows them to interact with different systems (such as emulsions, oily molecules, micelles and cellular membranes), which would favor their use in processed foods, as vehicles for drugs, antimicrobial agents, antioxidants in the cosmetic industry and even in the treatment of degenerative diseases related to oxidative stress

## 1. Introduction

Polyphenols are the secondary metabolites of plants and are generally involved in defense against ultraviolet radiation or aggression by pathogens (abiotic and biotic factors). In food, polyphenols may contribute to sensorial and physicochemical characteristics such as bitterness, astringency, color, flavor, odor and oxidative stability [1]. From a chemical point of view, phenolic compounds present diverse structures; however, they can be defined as compounds that possess at least one aromatic ring and one or more hydroxyl groups [2]. Based on the number of hydroxylated aromatic rings and the type of functional moiety, phenolic compounds can be classified into two main classes know as flavonoids and non-flavonoids. On one hand, flavonoids as the class with the greatest diversity of compounds, are subclassified into flavonones, flavones, anthocyanins, isoflavones, flavonols and flavanols. Additionally, phenolic compounds can polymerize into larger molecules, such as condensed tannins (also known as proanthocyanidins). On the other hand, regarding compounds classified as non-flavonoids, a wide variety of phenolic derivatives have been found in foods, including simple phenols, phenylpropanoids, benzoic or cinnamic acid derivatives and stilbenes. Hydrolizable tannins, lignans and lignins are also included in this category [3,4,5,6,7,8,9,10].

The antioxidant and other biological functions of polyphenols are attributed to their chemical structure. The aromatic structure and the hydroxyl groups present in them make these compounds good donors of electrons and hydrogen atoms, neutralizing free radicals and other reactive oxygen species (ROS) responsible for oxidative stress reactions [11,12]. Most of the natural antioxidants extracted from plants exhibit the hydrophilic character of phenolic acids; therefore, improving the solubility of phenolic acids and other phenolic compounds would allow the extension of their use as antioxidant additives in oils, fats and emulsions [13].

**Figure 1:**
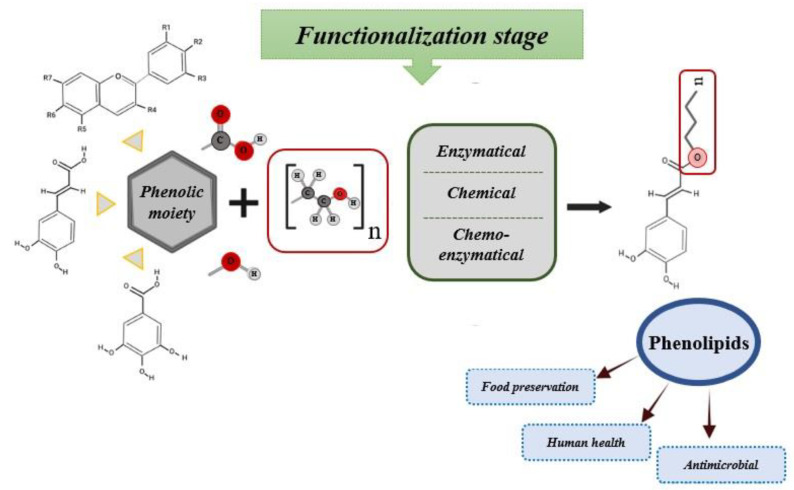
Graphical abstract of synthesis of phenolipids.

Phenolic compounds modified with aliphatic chains by lipophilization are named phenolipids or lipophenols and are capable of acting as oil soluble free radical scavengers [13,14,15,16] (Figure 1). The health benefits of phenolipids are attributed to the characteristics of the parent polyphenolic compound, since it is the phenolic motif present in amphipathic phenolipids that preserves their beneficial antioxidant and biological effects. The lipid domain confers special physicochemical properties to the phenolipids, allowing interaction with polar compounds with phenolic aglycone and can also react with nonpolar compounds through their hydrophobic moiety [15].

The lipophilization of phenolic acids through esterification or transesterification with fatty alcohols has been a simple and effective strategy to carry out the synthesis of amphiphilic antioxidants (namely phenolipids), which can be used in oil-based formulas, as well as in micellar, emulsified and liposomal systems [16,17]. Phenolic esters modified with a grafted fatty acid acyl chain have been observed to modify the antioxidant properties of the parent compound. Moreover, they have shown improved metabolic stability and bioavailability due to their higher lipophilicity with respect to corresponding phenolic parent compounds that undergo fast transformation and elimination in humans, mainly due to their hydrophilic character [14].

With this review, it is intended to summarize the information available around the functionalization of phenolic compounds by lipophilization with either saturated and unsaturated fatty alcohols or acids, and also with acyl chlorides, with the aim of modifying their natural solubility by transforming them into an amphipathic molecule. These modifications can enhance their inherent antioxidant and protective capacity as well as the biological activity of the parent phenolic compounds, allowing phenolipids to be able to establish interactions in the hydrophobic environment. Additionally, a review of some research work carried out to evaluate the performance of phenolipids in application areas such as processed foods, antimicrobials and human health will be presented.

## 2. Synthesis of Phenolipids

**Figure 2:**
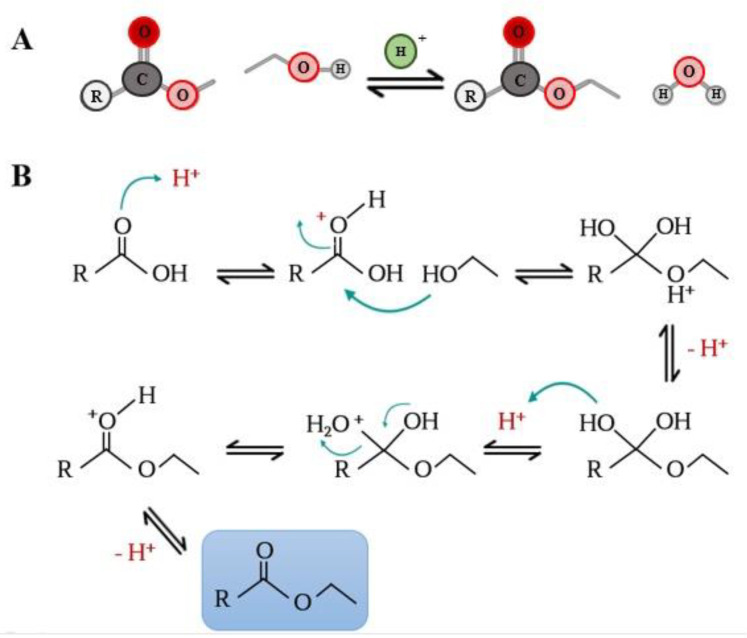
Fischer Esterification: (**A**) general esterification reaction by the Fischer method, which consists of the coupling between a carboxylic acid and an alcohol in the presence of an acid catalyst. (**B**) esterification reaction mechanism, in which a proton is transferred to the carbonyl oxygen; subsequently, the carbon undergoes a nucleophilic attack that leads to the transfer of the proton and finally to the formation of the ester with loss of water.

**Figure 3:**
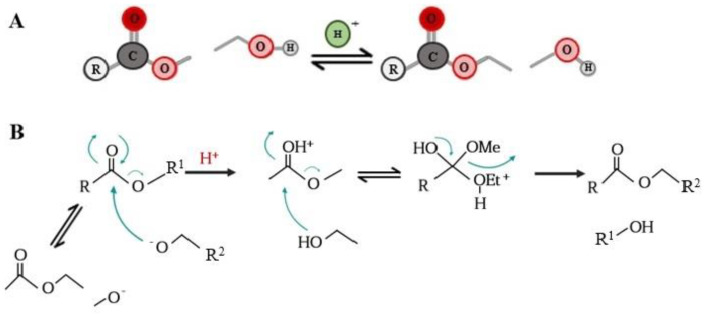
Transesterification. (**A**) general transesterification reaction, which consists of the process of exchanging the alkoxy group of an ester with another alcohol. The reaction is catalyzed by the addition of an acid that protonates the carbonyl group and makes the ester more electrophilic or by the addition of a base (that makes the alcohol more nucleophilic). (**B**) transesterification reaction mechanism, in which the carbon of the carbonyl group of the initial ester reacts to form a tetrahedral intermediate, which can revert to the initial compound or proceed to the transesterified product.

The functionalization of phenolic acids by attaching lipophilic moieties to a polyphenolic molecule (hydrophilic core) can be performed by chemical, enzymatical or chemo-enzymatical synthesis process, with the aim of esterifying the –COOH or the -OH residues present in the polyphenolic parent compound with fatty alcohols or acids (respectively) with aliphatic chains with variable length. Also, esterification reaction can be carried out using triacylglycerols or phospholipids. The chemical synthesis of phenolipids is a relatively quick and simple process, although not a selective process that also requires drastic reaction conditions such as extreme pH values and elevated temperatures, which favors the degradation of certain labile phenolic compounds under such conditions. In addition, the products obtained by chemical catalyst require purification steps with the aim of removing non-specific reaction products or solvent excess; therefore, the process becomes a long and expensive process that also generates polluting chemical residues. Due to the aforementioned, enzymatic synthesis using enzymes with hydrolase activity (such as lipases) for the esterification process is a more convenient option. Esterases such as lipases are known to catalyze esterification (reverse reactions) and transesterification in non- or low-aqueous solvent media [18,19,20,21]. Among the advantages of synthesis using enzymes with hydrolytic capacity are the generation of fewer residues, as well as a greater specificity of esterification and a reduction in the purification steps of the phenolipids obtained. However, enzymatic inhibition may occur due to the reaction conditions, such as incompatibility with some solvents or substrates, the pH of the medium and even the type of molecule to be modified [16,17,18,22,23,24]. Direct lipophilization reactions can be carried out in conjunction with a catalytic enzyme in the presence of different organic solvents or in a solvent-free system, where the fatty alcohol acts as both reagent and solvent. An alternative for the use of organic solvents in the synthesis process may be the use of ionic liquids or deep eutectic solvents (DES). These types of media offer a stable environment for the catalytic enzymes, avoiding their inhibition and enhancing their selectivity. Furthermore, the inherent characteristics of both ionic liquids and DES confer on them low toxicity and they are easy to dispose of; therefore, they are friendly to the environment [17,18,25]. Finally, an intermediate synthesis strategy consists of initiating the reaction by the chemical route to obtain the methyl ester of the phenolic acids by esterification (Figure 2) and to continue by the enzymatic route for transesterification with the fatty alcohol (Figure 3) [16].

In this section, the synthesis of phenolipids carried out by various experimental processes is presented as a table, in which are mentioned: (a) the type of polyphenolic molecules that are modified; (b) fatty alcohols or acids of various chain length, both saturated and unsaturated, or acyl chlorides; (c) the synthesis process and finally (d) the reaction media (Table 1).

Over time, several researchers have carried out the synthesis of phenolipids from polyphenols with a simple structure (mainly phenolic acids and/or their derivatives) and even from some flavonoids, obtaining good yields using the available and reported synthesis methods by other authors. In most cases, the post-synthesis steps involve the purification of the esters obtained by means of solid phase separation (SPS), liquid–liquid extraction, semi-preparative liquid chromatography and column chromatography, among other techniques, as well as their subsequent characterization and quantification by analytical methods such as high-performance liquid chromatography (HPLC), mass spectrometry (MS), gas chromatography (GC) and/or nuclear magnetic resonance (NMR).

## 3. Phenolipids and Antioxidant Capacity

Antioxidant assays represent an essential step in the evaluation of the antioxidant capacity of compounds obtained from natural sources, such as medicinal plants and certain foods [3]. The radical scavenging capacity of natural antioxidants can be classified as two major mechanisms: hydrogen atom transfer (HAT) and electron transfer (ET). Hydrogen atom transfer behavior, in the case of phenolic compounds, is related to an antioxidant compound with the capacity to transfer hydrogen protons to the lipid radicals in order to stop the propagation step of the lipid oxidation pathway [15,50].

Natural antioxidants such as polyphenolic compounds that possess hydrogen atom transfer capacity are named free radical scavengers (FRS). Phenolics are effective FRS because phenolic free radicals have low energy due to the delocalization of the free radical throughout the phenolic ring structure. Each FRS is capable of inactivating at least two free radicals, the first being inactivated when the FRS interacts with the initial oxidizing radical and the second when the FRS radical interacts with another radical via a termination reaction to form a nonradical product [15].

However, even though the functionalization of phenolic compounds for the synthesis of phenolipids seems to improve the antioxidant capacity compared to the parent polyphenolic compound, it has been observed by different researchers that the length of the alkyl chain seems to be involved in the improved behavior of phenolipids. The experimental results of various authors show that, with respect to the size of the alkyl chain of the synthesized phenolipids, there is a breaking point called the “cut-off effect”, in which the phenolipids lose their particularly improved behavior, which is reflected in a decrease in antioxidant capacity and reduction of biological effects (for example, antimicrobial activity).

Moreover, it has been observed that the functionalization of polyphenols does not necessarily favor an increase in antioxidant capacity or in the interactions in heterogeneous systems (for example, emulsions). This is reflected in the experimental results of the quantification of the antioxidant capacity of the phenolipids synthesized from polyphenols (mostly phenolic acids), in which a lower behavior with respect to the parent polyphenolic compound (without functionalization) has been observed. However, another factor that influences the antioxidant capacity of phenolipids is the type of modified polyphenolic core, which is directly responsible for the interruption in oxidative processes depending on the amount and position of the -OH groups available, the type of functional residue that they possess or the conformational characteristics of the polyphenolic molecule. Finally, it is important to mention the breaking point with respect to the attached hydrophobic chain length (cut-off effect), which seems to have a direct influence on the behavior of the phenolipids and the amplification or the decrease in their antioxidant capacity, due to the location that allows it to occupy hydrophobic character systems, and the types of interactions between phenolipids and the components present in the surrounding environment.

In this section, the evaluation of the antioxidant capacity of phenolipids by the radical scavenging method is summarized as a table, in which are mentioned: (a) the type of phenolipid; (b) the parent polyphenolic compound; (c) the length of the attached fatty chain and finally (d) the comparison of the radical scavenging behavior determined by the 2,2-diphenyl-1-picrylhydrazyl radical (DPPH) assay between the parent compound and modified derivates (Table 2).

## 4. Potential Applications of Phenolipids

Polyphenols, due to their proven ability as a free radical scavenger, metal chelator and disruptor of chain oxidation processes, have been a subject of interest in various fields such as food preservation, cosmetics, pharmacology and dermatology among others. Due to their nature, they present limitations for their full use, highlighting the low solubility in oily matrices due to their hydrophilic behavior, which prevents the location of the phenolic motif in the right place to carry out its protective function. However, a growing strategy for the functionalization of polyphenols consists of the synthesis of phenolipids derived from the addition of hydrophobic fatty acid chains to the phenolic core. Phenolipids exhibit amphiphilic behavior, which gives them physicochemical properties to be able to interact, for example, in micelles present in emulsion systems, drug-transporting vesicles and even with biological membranes.

It is important to mention that the biological effects (antimicrobial, anti-inflammatory, antiparasitic, anticancer among others) and antioxidant capacity in phenolipids are directly linked to the type of phenolic compound of the hydrophilic part of the functionalized molecule. Therefore, the length of the lipophilic chain determines the capacity for interaction with other hydrophobic molecules, as well as its distribution and location, for example, at the interfacial region in the presence of surfactant agents in oil-in-water (o/w) emulsion systems or among the lipid bilayer of cells with the hydrophilic core oriented towards the inner. The main challenge in the synthesis of phenolipids and their possible fields of application lies in finding the ideal hydrophobic chain length to guarantee its protective function, as well as avoiding the “cut-off effect”, in which phenolipids lose their improved performance due to the length of the lipophilic chain.

In order to explore the advances in phenolipids and their application, in this section a brief review of some research work carried out by several authors is presented, with the aim of exemplifying the challenges and limitations observed in various fields of application.

### 4.1. Preservation and Stability in Food

The metabolism of atmospheric oxygen produces highly reactive oxygen species (ROS) that can cause the oxidation of food constituents, including lipids, pigments, vitamins and proteins. The degradation process in food initiated by oxygen reactivity has the consequences of off-flavor formation, loss of color and the transformation of important nutrients, which decrease the quality of the food and are risk factors for the development of diseases [9,15,33]. Therefore, a dietary antioxidant can scavenge reactive oxygen/nitrogen species (ROS/RNS) to stop radical chain reactions, or it can inhibit the reactive oxidants from being formed in the first place (preventive). Dietary antioxidants often broadly include radical chain reaction inhibitors, metal chelators, oxidative enzyme inhibitors and antioxidant enzyme cofactors [50].

The control of lipid oxidation is important in the continuing development of new strategies for preparing foods with higher nutritional profiles and longer shelf lives. In order to retard or inhibit the oxidative process, the addition of an antioxidant agent has been the strategy mainly adopted and the oxidative stability of food lipids, as proof of antioxidant efficiency, is usually estimated by determining the extent of oxidation under a set of standardized conditions [36,37,50,51]. In emulsified systems, the ability of antioxidants to inhibit lipid oxidation depends on diverse factors, such as (a) the antioxidant concentration reactivity; (b) the partitioning between oil, water and interfacial regions; (c) the interactions with other components such as emulsifiers; (d) other antioxidants and (e) environmental conditions (pH, ionic strength and temperature, among others) [37].

Lipid oxidation is suggested to be initiated at the interface between the oil phase and the aqueous phase or air, and subsequently continues in the oil phase, starting its decomposition [53]. Lipophilic or hydrophilic antioxidants in an edible form and considered safe are usually employed to stabilize a wide variety of oil-enriched foods [54]. Foods containing long-chain polyunsaturated fatty acids (PUFAs) (for example, enrichment food with docosahexanoic acid -DHA) are especially labile with respect to oxidation, which causes the formation of undesirable flavors and rancid odors, the production of potentially toxic compounds and the loss of the health-beneficial and essential fatty acids [29].

In order to verify the antioxidant effect of phenolipids synthetized from phenolic acids in foods containing oils and fats, various authors have carried out investigations especially in emulsified systems. Sørensen et al. (2012) [55] evaluated the antioxidative effect of C8/C18:1 dihydrocaffeates and C12/C16 rutinates in fish oil-enriched milk emulsions. Also, parent polyphenolic compounds were tested. Among the different phenolipids tested, the C12 rutin ester was the most efficient antioxidant in inhibiting the formation of peroxides (PV) in milk emulsified with fish oil and acetone, followed by dihydrocaffeic acid, C18:1 dihydrocaffeate, rutin and finally C16 rutin palmitate. Moreover, in milk emulsified with fish oil without acetone, the C8 dihydrocaffeate presented the best protective profile, followed by C18:1 dihydrocaffeate, caffeic acid and dihydrocaffeic acid. In the case of volatile inhibition behavior, in milk emulsified with fish oil and acetone, rutin laurate followed by oleyl dihydrocaffeate were the most efficient antioxidants compared to other phenolipids and their parent compounds. For milk emulsified with fish oil without acetone, C18:1 dihydrocaffeate and C8 dihydrocaffeate showed the best results. In a similar study, Alemán et al. (2015) [52] also proved the antioxidative effect of lipophilized caffeic acid in milk and mayonnaise both enriched with fish oil. In mayonnaise, caffeic acid and caffeates C1–C18 and in milk caffeic acid and caffeates C1–C20 were evaluated as antioxidants during storage. Fish oil-enriched mayonnaise with caffeates of medium alkyl chain length (C4, C8 and C12) presented the best protective behavior, whereas in fish oil-enriched milk emulsions the most effective caffeates were those with shorter alkyl chains (C1 and C4). Finally, Qiu *et al*. (2017)[56] evaluated the antioxidant effect of ferulic acid and its C1, C2 and C12 ferulates (and their mixes) in fish oil-enriched milk (eicosapentaenoic acid (EPA, 20:5) and docosahexaenoic acid (DHA, 22:6)). The C1 and C2 ferulates showed a very slight increase in PV throughout the storage period (13 days) and had the lowest PV compared to those of all the other treatments. The C1/C12 and C2/C12 ferulates had very similar, slightly increasing PVs over the whole storage period, in agreement with the very similar PVs of the C1 and C2 ferulates. Regarding the quantification of the volatile compounds generated as secondary oxidation products during the 13 days of storage, both the C1 and C2 ferulates presented the best protective profile against the formation of 1-penten-3-one, hexanal, 1-penten-3-ol and 2,4- heptadienal.

In the case of prepared food, Szydłowska-Czerniak and Rabiej (2021) [10] evaluated the effect on frozen French fries fried with rapeseed oil enriched with C8 sinapate, ferulate and caffeate, in addition to C16 sinapate and ferulate. Once the frozen potatoes were fried, the oils (enriched and without phenolipids) were analyzed to determine the formation values of peroxide, anisidine (p-AnV), free fatty acid content (AV), conjugated diene (CD) and conjugated triene (CT). The antioxidant capacity and total polyphenolic content (TPC) were also measured by ABTS, DPPH, FRAP and Folin–Ciocalteu, respectively. The addition of phenolipids to rapeseed oils before the frying process caused an increase in the amounts of hydroperoxides (PV). However, after the heat treatment the changes in PV during the frying of the French fries depended on the type of phenolipid added to the oil, whereas oils with C16 ferulate and C8 sinapate oxidized at a slower rate. Moreover, the addition of phenolipids to rapeseed oils did not significantly affect the CD values of the unheated and heated French fries. The highest inhibitory effect on the formation of secondary oxidation products during frying determined as p-AnV was observed in the C16 and C8 sinapate. With respect to antioxidant capacity, the FRAP and DPPH of the oil with phenolipids changed significantly during the frying process maybe due to the thermal treatment, whereas the ABTS of the oils with C8 caffeate and C16 sinapate did not differ significantly before and after frying. From a nutritional point of view, French fries fried in the enriched oils presented both higher antioxidant capacity and polyphenolic content than those prepared in the refined rapeseed oil without phenolipids.

### 4.2. Antimicrobial Capacity against Foodborne Pathogens

Chemically, food products consist of water, fat, carbohydrates, protein and small amounts of organic compounds and minerals. Thus, they will promote the growth of microorganims since all these compounds are the source energy for microbes to grow [57]. Phenolic compounds, particularly phenolic acids, have become an increasing source of interest for the food industry stemming from their potential bioactive properties. Besides, plant phenolics and extracts rich in such substances can be excellent inhibitors of many foodborne pathogenic and spoilage bacteria activities [53,58].

Aissa *et al*. (2012) [47] probed the inhibitory spectrum of synthetized phenolipids from tyrosol (esters with chain length from C1 to C18) against eight Gram-positive bacteria and five Gram-negative bacteria with pathogenic behavior in humans, as well as their activity against two strains of promastigotes of the *Leishmania* sp.. Regarding antimicrobial activity, the tyrosyl esters of C8, C10 and C12 chain length showed an effect on three Gram-positive and one Gram-negative microbial strain. The antileishmanial activity of the tyrosol derivatives was observed at the same range of chain length (C8, C10 and C12) as the antimicrobial effect described above. The authors concluded that the C10 tyrosyl ester was the most effective behavior and determined as the maximal chain length with biological activity (cut-off effect). Within the same line of evaluation, Shi *et al*. (2021) [58] evaluated in vitro the antimicrobial effect of C1, C4,C6, C8, C10, C12 and C14 gallates against *Escherichia coli* and *Staphylococcus aureus*. The results showed that gallates with a longer alkyl chain length exhibited an increase in antibacterial activity. In the case of the *E. coli* strain, the C8 gallate presented the best bactericidal activity, followed in decreasing order of effectiveness by C6, C10/C12 and finally C14/C4/C2 gallates along with gallic acid (parent compound). In the *S. aureus* strain, the C8/C10 and C12 gallates showed the principal biological effect, followed in decreasing order of effectiveness by gallic acid and C2/C4 gallates, C14 and C6 gallates. The microbiological bacteriostatic concentration (MBC) established for the *E. coli* strain showed that the C8 gallate showed a better value among the other phenolipids evaluated, and for the *S. aureus* strain C10 and C12 gallates presented the same behavior. The authors concluded that the C8 gallate functions against both bacteria through damaging bacterial cell wall integrity, permeating into cells and then interacting with DNA, as well as disturbing the activity of the respiratory electron transport chain to induce a high-level toxic ROS (hydroxyl radicals) generation and up-regulation of the ROS genes. Also, *Shi et al*., in their study (2018a, 2018b) [53,58], evaluated the bacteriostatic and bactericidal capacity of C1-C18 ferulates against both *E. coli* and *Listeria monocytogenes*. For both pathogens, C6 ferulate was the most effective bactericidal agent through various mechanisms such as membrane disruption, altering cell morphology and possibly anchoring to bacterial DNA-forming complexes. In addition, Zieniuk *et al.* (2021) [30] evaluated the efficiency of five (C2, C4, C6 C8 and C10) esters of 4-hydroxyphenylpropanoic acid against *L. monocytogenes* PCM 2191. The phenolipids were obtained in reactions catalyzed by *Candida antarctica* lipase B and different doses were probed to determine the microbiological inhibitory concentration (MIC) and microbiological bacteriostatic concentration (MBC). The C8 4-hydroxyphenylpropanoate was the most active compound against *L. monocytogenes* PCM 2191 as a bacteriostatic and bactericidal compound. The authors concluded that the esterification of phenolic acid allowed the obtaining of more active compounds compared to their parent compounds, which is related to increased lipophilicity that favors the interaction with the pathogen membrane and thus exerts the expected biological effect.

### 4.3. Health Benefits

One of the main causes of health imbalance is related to oxidative stress, causing cell affectation because of their inability to stop damage by neutralizing free radicals, leading to numerous degenerative and chronic diseases [3,9,11,59]. The polyphenols present in the diversity of foods available in the human diet have demonstrated their capacity as antioxidant and protective agents against various degenerative diseases related to the imbalance between oxidative processes inherent to the cellular metabolism and mechanisms (endogenous and exogenous). However, because of their inherent behavior in lipid matrices, coupled with the low solubility that some phenolic compounds present, the functionalization of polyphenols by the grafting of aliphatic chains seems to be a viable strategy for their use in various fields.

Phenolipids, as amphipathic molecules, present better miscibility and incorporation into lipid phases and lipocarriers, and offer an advantage for their use in drug delivery systems, pharmaceuticals, nutraceuticals, foods and cosmetic formulations [17]. Some studies have been carried out with the aim of evaluating the impact of functionalized polyphenols as phenolipids and the inherent biological effects of parent polyphenolic molecules. The natural solubility of polyphenols limits the free passage through hydrophobic cell membranes of both pathogenic and food spoilage microorganisms, as well as human cells. Therefore, when modifying polyphenols to obtain amphipathic phenolipids, it is expected that the interaction capacity of phenols with hydrophobic molecules will increase thanks to the esterified aliphatic motif as a functionalization strategy. Additionally, phenolipids in aqueous medium (and depending on the length of the aliphatic chain), may associate for the formation of micelles, which increase the diffusion capacity through cell membranes, allowing the presence of the antioxidant core (polyphenolic compound) in the cytosolic space.

As a strategy to combat cardiovascular diseases, Chalas *et al.* (2001) [27] evaluated the inhibitory effect of the C2 phenolipids of gallic, caffeic, synaptic, ferulic and *p*-coumaric acids in copper-induced low-density lipoprotein (LDL) oxidation and the hemolysis induction of erythrocyte membranes by the AAPH method. On one hand, their results showed a protective behavior of phenolipids against the modifications of fraction A of LDL obtained by separation on an ion exchange column. On the other hand, the phenolipids also inhibited the increase in the negative charge fraction of the LDL molecules because of Cu^2+^-catalyzed oxidation in a concentration-dependent manner. All the C2 phenolipids were more potent than their corresponding parent compounds. The C2 caffeate was the most potent phenolic acid ester, followed by C2 sinapate and ferulate and finally C2 gallate and p-coumarate, which presented the weakest effect. Regarding the protective effect against hemolysis in the AAPH assay, the caffeic and gallic C2 esters were more efficient than their parent phenolic compounds as C2 ferulate (with less intensity than the previous ones), while C2 sinapate and p-hydroxy cinnamate had unchanged activity, compared to their corresponding non-modified phenolics acids. In the same line of research, a similar protective behavior on LDL stability was observed by Katsoura *et al.* (2009) [28] when evaluating the antioxidant capacity *in vitro* of three phenolipids synthetized from ferulic acid (C1, C2 and C8 ferulates) on LDL, HDL (isolated from freshly prepared human plasma) and total serum in the presence of copper (as oxidation inductor). The results showed that the C8 ferulate obtained the better antioxidant effect, attributing its efficiency to the length of the alkyl chain. In conclusion, the modification of ferulic acid by lipophilization can enhance its biological effect as an antioxidant protector of the *in vivo* LDL oxidation process, able to prevent the early development of atherosclerosis.

In the particular case of evaluating the diffusion and the intake capacity of amphipathic phenolipids in human cells as well their respective antioxidant effect, Bayrasy *et al.* (2013) [60] lipophilized rosmarinic acid with various aliphatic chain lengths (C4, C8, C10, C12, C16 and C18) to synthesize rosmarinate alkyl esters and evaluated in modified human dermal fibroblasts overexpressing their *in vitro* ability to reduce the level of reactive oxygen species (ROS), to cross fibroblast cell membranes and to target mitochondria. Their results showed that C10 rosmarinate presented the better antioxidant activity, and a decrement was observed in phenolipids with longer chain length. Moreover, the C4, C8 and C10 rosmarinates passed through the membranes, whereas longer phenolipids were not able to cross membranes and formed extracellular aggregates. Besides cell uptake, the alkyl chain length also determined the subcellular localization of rosmarinates. In the case of mitochondria, medium chain-length rosmarinates were observed, as well as short chain-length rosmarinates in the cytosolic space and finally, extracellular media for longer chain-length rosmarinates.

For the evaluation of the effect of phenolipids with respect to degenerative diseases related to oxidative stress, such as cancer, Fiuza *et al.* (2004) [38] carried out the synthesis of the phenolipids of caffeic and gallic acids using methanol, n-propanol and n-octanol with the aim of evaluating their antiproliferative and cytotoxic effects in the human cervix adenocarcinoma cell line-HeLa by MTT assay, the Trypan blue exclusion method and Alamar blue assay. In addition, the authors correlated the biological effect obtained in HeLa and L-132 (non-neoplastic cell–fibroblasts from human embryonic lung tissue, as experimental control) cells with the conformational structure of each caffeate and gallate synthetized. The results showed that the cytotoxic activity of the phenolic derivatives tested seemed to be strongly dependent on their structure and the variation in the length of the alkyl chain. The C3 caffeate and gallate displayed considerably more pronounced antiproliferative and cytotoxic effects towards both the HeLa and L-132 cell lines than their C8 and C1 caffeates and gallates. In conclusion, the authors observed that the presence and number of -OH ring substituents in phenolipids is a determinant of the corresponding biological activity. Accordingly, for the same length of the alkyl chain, the trihydroxylated polyphenolic esters displayed a higher antiproliferative and cytotoxic effect than the dihydroxylated ones.

In the same way, Li *et al.* (2009) [54] carried out the synthesis of seven phenolipids starting from ferulic acid under a microwave irradiation procedure, with different chain lengths (C1, C2, C3, C4, C5, isopropyl, isobutyl and isopentyl alcohols were used). Subsequently, Li *et al.* (2012) [61] evaluated the antiproliferative and cytotoxic effects of the ferulates previously synthesized by Li *et al.* (2009) [54], in addition to carrying out the synthesis of caffeates. The biological tests were carried out using 14 cell lines from various types of human cancer: A549, H157,1299, H460, Calu 1, 1792, H266, Hop62 and 292G (lung cancer); LOX-IMVI and M14 (melanoma); HeLa (cervical cancer); M4E (neck and head cancer) and SKBR (breast cancer). The anticancer activity was variable in concordance with the type of polyphenolic core (ferulic or caffeic acid) and the lipophilic chain length. The phenolipids of C3/C4 ferulic and caffeic acid (synthesized from propyl and butyl alcohols) showed an effect on the 14 cell lines, being the phenolipids with ferulic acid as parent compound; those with less anticancer activity (such as C5 ferulate) did not show a significant effect against any cell line). The C1 ferulate did not show a significant effect in 13 of the 14 cell lines, except in the HeLa line of cervical cancer. The C1 caffeate presented the opposite effect, except in the H266 line of lung cancer. In the case of C2 ferulates and caffeates, no significant effect was observed on melanoma cells or on the H266 line of lung cancer, respectively. The isopropyl ferulates were effective only in HeLa cells and isopropyl caffeate only on three lung cell lines (H157, H46 and 1299), and on cervical HeLa cells and breast cancer SRKB cells. Similarly, in the case of isobutyl ferulates, the observed effect was presented in four lung cells lines (A549, H157, H266 and 1299), cervical and breast cancer cells. On the other hand, isobutyl caffeate showed an effect on five lung cancer cells, besides cervical, neck/head and breast cells. Finally, only the isopentyl ferulate did not present an effect on HeLa cells. The results obtained by the authors support the possible therapeutic use of phenolipids synthesized from ferulic and caffeic acids due to the spectrum of biological action shown in various lines of cancer, particularly in the HeLa cervical cancer line, which globally showed increased sensitivity to the phenolipids of various chain lengths (both ferulates and caffeates).

Relating to neurodegenerative diseases related to the imbalance between oxidative species and the mechanisms that regulate them, Jantas *et al.* (2020) [62] studied the neuroprotective effect of C1 caffeate in differentiated and undifferentiated neuroblastoma SH-SY5Y cells and primary neuronal cell cultures from mouse C57Bl/6J embryos. Moreover, the authors analyzed the cytotoxic effect by lactate dehydrogenase (LDH) release assay, cell viability by MTT assay and apoptosis activation caused by methyl caffeate in both cell cultures. A protective effect of C1 caffeate against hydrogen peroxide damage by oxidative stress was observed in both undifferentiated and differentiated SH-SY5Y, as well in primary neuronal cells. Moreover, the inhibition of the cell death process induced by neurotoxicity was detected in differentiated SH-SY5Y cells treated with methyl caffeate. Additionally, an antiproliferative effect was observed in undifferentiated SH-SY5Y cells treated with the higher doses tested of C1 caffeate and presented a sensitizing effect on cells when combined with a chemotherapeutic agent.

Finally, Kaihatsu *et al.* (2009) [63] probed the cytotoxicity effect of C16 EGCG (epigallocatechin gallate) mono-ester by the MTT cell proliferation assay on MDCK (Madin Darby Canine Kidney) cells infected with a series of human influenza viruses, an experimental strain (A/Puerto Rico/8/34/(H1N1)), vaccine strains [A/Beijing/262/95/(H1N1), A/Panama/2007/99/ (H3N2) and B/ Yamanashi/166/98/], drug-resistant strains ([Yokohama/77/2008/(H1N1)) OPR: oseltamivir phosphate-resistant (OPR), Yokohama/63/2007/ (H1N1) AR: amantadine-resistant (AR), A/Yokohama/91/2008/(H1N1) OPR/AR: (OPR/\\AR)) and avian pathogenic influenza (A/Duck/Hong Kong/342/78/(H5N2)). In addition, the authors evaluated the protective behavior of the ECGC phenolipid using chicken-embryonated eggs inoculated previously with C16 ECGC mono-ester and subsequently infected with the avian H2N2 influenza virus. Their results showed in both evaluations that C16 EGCG phenolipid offered a clearly antiviral activity against human respiratory human viruses and it could be used as a protector agent (in this case) for the avian population against H2N2 virus attack. Similar studies were carried out by Zhong, Ma and Shahidi (2012) [45], who evaluated ECGC phenolipids with saturated (stearic and octyl butyrate acid) and polyunsaturated fatty acids (eicosapentanoic and docosahexaenoic acids) attached as the lipophilic domain, where those with an unsaturated chain showed a marked antiviral activity against the hepatitis C virus, due to their protease inhibitor capacity. Besides the above, the authors reported an *in vitro* α-glucosidase inhibitor activity from stearic acid ECGC phenolipid compared with acarbose; therefore it could be used as a treatment against the HIV virus due to the blockade of the glycosylation of the viral envelope glycoproteins (infection step) and eventually in type 2 diabetes management.

## 5. Conclusions

Polyphenols, mainly as antioxidant additives of natural origin, provide processed foods with additional dietary benefits and contributions to consumer health due to their biological effect. However, since they are highly polar, their incorporation into matrices with fatty acids limits their protective capacity. The functionalization of phenolic molecules through esterification with alcohols and fatty acids provides them with favorable characteristics for their interaction in heterogeneous systems. Since the degradation of the constituent compounds of food represents a risk to human health, the use of phenolipids as a disruptor of oxidative processes seems to offer an alternative for their safe and efficient use. However, the performance of phenolipids will depend on the type of polyphenolic species that constitutes the hydrophilic core, while the length of the lipophilic domain allows it to interact with other fat molecules or even self-complex to form micellar or vesicular structures. This ability would favor the passage through cell membranes, which would allow the correct localization of polyphenols to carry out their biological effect. Either by means of chemical or enzymatic synthesis, the modification of polyphenols by esterification represents a functional alternative with wide potential to replace the synthetic antioxidants that are generally used in oily matrices. Within the limitations of its use in various areas is the optimization of the appropriate aliphatic chain length. However, standardization results are contradictory since there is evidence of variations in the protective performance of phenolipids depending more on the type of parent polyphenolic core but affected by the length of the lipophilic chain. Since the lipophilic domain of phenolipids does not contribute directly to the characteristics of polyphenols, but rather allows them to maintain interactions in regions of less hydrophilic character, the greatest challenge lies in finding a balance in the design of widely functional phenolipids.

## Figures and Tables

**Table 1:** Diversity of phenolipids synthetized by several authors.

Polyphenolic Family	Phenolic Core	Lipophilic Domain	Synthesis Procedure	Functionalization Reaction Media	Yield	References
HBZ-PA	Gallic acid	C6	Enzymatic	Diethyl ether	<2%	[26]
		C2	Chemical	Data not available	Data not available	[27]
HBZ-PA	*p*-hydroxybenzoic acid	C6	Enzymatic	Diethyl ether	<2%	[26]
HBZ-PA	Syringic acid	C6	Enzymatic	Diethyl ether	<2%	[26]
HBZ-PA	Vanillic acid	C6	Enzymatic	Diethyl ether	<2%	[26]
HBZ-PA	3,5-di-*t*-butyl- 4-hydroxybenzylalcohol	C8	Enzymatic	Cyclohexane	98%	[26]
HBZ-PA	*p*-Hydroxyphenyl acetic	C1 to C10	Enzymatic	1-ethyl-3-methylimidazolium hexafluorophosphate	13.4–62.6% (C8)	[28]
HBZ-PA	Protocatechuic acid	C1, C2, C3	Chemical	Fatty alcohols	71–81%	[29]
		C1, C3,C6,C8, C10, C12, C14, C16, C18	Chemical	Fatty alcohols	59–95%	[19]
HBZ-PA	Gentisic acid	C6	Enzymatic	Diethyl ether	<2%	[26]
HBZ-PA	4-hydroxyphenylpropanoic	C2, C4, C6, C8, C10	Enzymatic	Methyl-*tert*-butyl ether, fatty alcohols	Data not available	[30]
HCM-PA	Ferulic acid	C2, C8	Enzymatic	2-methyl-2-propanol toluene/hexane	14–20% 50% (C8)	[31]
		C18 (linoleyl alcohol)	Enzymatic	Hexane, 2-butanone	16%	[32]
		C2	Enzymatic	Isooctane	87%	[33]
		C4, C8, C12, C16	Chemo- enzymatic	Methanol, ChCl-urea-water system, 1-alkanol	98%	[34]
		C1 to C10	Enzymatic	1-ethyl-3-methylimidazolium tetrafluoroborate/hexafluorophosphate,1-butyl-3-methylimidazolium tetrafluoroborate/ hexafluorophosphate, 1-octyl-3-methylimidazo methylimidazolium tetrafluoroborate/ hexafluorophosphate	6.7–62.6% (C8, [emim]PF6) 2.3–4.9% (C4, [emim]BF4) 3.4–14.9 (C4, [bmim]BF4) 7.6–18.2 (C4, [omim]BF4) 23.4–34.1 (C4, [emim]PF6) 32.9–52.6 (C4, [bmim]PF6) 55.6–59.2 (C4, [omim]PF6) 3.9–7.3 (C4, [bmim]BF4/[bmim]PF6 90/10) 10.2–12 (C4, [bmim]BF4/[bmim]PF6 50/50) 31.3–31.5 (C4, [bmim]BF4/[bmim]PF6 10/90)	[28]
		C2, C4, C8, C12, oleyl alcohol	Enzymatic	Fatty alcohols	Traces (C4-C8) 2% (C12-oleyl alcohol)	[22]
		Oleyl alcohol	Enzymatic	*tert*-butanol isooctane toluene 2-butanone hexane cyclohexane 1-butyl-3-methylimidazoliumbis (trifluoromethylsulfonyl) mide/ isooctane 1-hexyl-3-methylimidazolium hexafluorophosphate/ isooctane 1-methyl-3-octylimidazolium hexafluorophosphate/ isooctane 1-butyl-3-methylimidazolium hexafluorophosphate/ isooctane 1-butyl-3-methylimidazol umtetrafluoroborate/ isooctane	3.81% 97.68% 25.83% 0% 99.17% 74.02% 16.51 mg/mL 14.67 mg/mL 12.53 mg/mL 4.03 mg/mL 2.37 mg/mL	[35]
		C2	Chemical	Data not available	Data not available	[27]
		C1, C4, C8, C12, C16, C18, C20	Chemical	THF, sulfuric acid, ethyl acetate	>90%	[13]
HCM-PA	*p*-coumaric acid	C4, C8, C12, C16	Chemo-enzymatic	Methanol, ChCl-urea-water system	98%	[34]
		C2	Chemical	Data not available	Data not available	[27]
HCM-PA	Cinnamic acid	C4, C6 and C12	Enzymatic	*n*-pentane, cyclohexane Diethyl ether, *t*-butylmethyl ether, 1-butanol	>85% (C4) 68–70% (C6-C12) <6%	[26]
		C1 to C10	Enzymatic	1-ethyl-3-methylimidazolium hexafluorophosphate	54.2–56.2% (C8)	[28]
		C2, C4, C8, C12, oleyl alcohol	Enzymatic	Fatty alcohols	30% (C4-C8-oleyl alcohol) 26% (C12)	[22]
HCM-PA	Caffeic acid	C1 to C10	Enzymatic	1-ethyl-3-methylimidazolium hexafluorophosphate	8.4–11.6% (C8)	[28]
		C1, C2, C3, C4, C6, C8, C10,C12, C14, C16	Chemical	Methyl, ethyl, propyl, butyl, hexyl, octyl, decyl, dodecyl tetradecyl and hexadecyl malonates, 3,4-dihydroxybenzaldehyde	Data not available	[36]
		C8, C16	Chemical	Monooctyl/ monohexadecyl malonates, 3,4-dihydroxybenzaldehyde	Data not available	[37]
		C2, C4, C8, C12, oleyl alcohol	Enzymatic	Fatty alcohols	0%	[22]
		C2	Chemical	Data not available	Data not available	[27]
		C2, C3, C8	Chemical	Fatty alcohols, sulfuric acid, dimethylformamide, ethyl ether, oxalyl chloride	Data not available	[38]
		C1, C4, C8, C12, C16, C18, C20	Chemical	THF, sulfuric acid, ethyl acetate	>90%	[13]
HCM-PA	Sinaptic acid	C1 to C10	Enzymatic	1-ethyl-3-methylimidazolium hexafluorophosphate	0.4–31.2% (C8)	[28]
		C2	Chemical	Data not available	Data not available	[27]
		C18 (linoleyl alcohol)	Enzymatic	Hexane, 2-butanone	99%	[32]
HCM-PA	Dihydrocaffeic acid	C8, C16	Chemical	Monooctyl/ monohexadecyl malonates, 3,4-dihydroxybenzaldehyde	Data not available	[37]
		C2, C4, C8, C12, oleyl alcohol	Enzymatic	Fatty alcohols	50% (C4), 38% (C8), 30–31% (C12-oleyl alcohol)	[22]
		C8	Enzymatic	Trioctylmethylammonium trifluoroacetate	62%	[39]
HCM-PA	Rosmarinic acid	C1, C4, C8, C12, C18 and C20	Chemical	Fatty alcohols	98.5% (C1) 99.3% (C4) 99.5% (C8) 4.4% (C12) 81.6% (C16) 99.0% (C18–20)	[40]
		C4, C8, C12, C18 and C20	Chemical	Fatty alcohols	Data not available	[12]
HCM-PA	Phloretic acid	C1 to C10	Enzymatic	1-ethyl-3-methylimidazolium hexafluorophosphate	38.5–60.6% (C8)	[28]
HCM-PA	4-hydroxicinnamic acid	C8, C16	Chemical	Monooctyl/ monohexadecyl malonates, 3,4-dihydroxybenzaldehyde	Data not available	[37]
HCM-PA	2,4 dihydroxihydrocinnamic acid	C1 to C10	Enzymatic	1-ethyl-3-methylimidazolium hexafluorophosphate	23.3–27.7% (C8)	[28]
HCM-PA	3,4 dihydroxihydrocinnamic acid	C1 to C10	Enzymatic	1-ethyl-3-methylimidazolium hexafluorophosphate	30.0–35.5% (C8)	[28]
HCM-PA	3,4 dimethoxy cinnamic acid	C2, C4, C8, C12, oleyl alcohol	Enzymatic	Fatty alcohols	60% (C4) 12% (C8) 10% (C12-oleyl alcohol)	[22]
HCM-PA	Clorogenic acid or isomers	C1, C4, C8, C12, C16, C18 and C20	Chemo-enzymatic	Fatty alcohols	Data not available	[24]
		C1, C4, C8, C12, C16, C18 and C20	Chemo-enzymatic	Fatty alcohols	Data not available	[41]
HCM-PA	*p*-methoxycinnamic acid	2-ethyl-hexanol	Enzymatic	Isooctane	90%	[33]
HCM-PA	Coumaric acid	C1, C4, C8, C12, C16, C18, C20	Chemical	THF, sulfuric acid, ethyl acetate	>90%	[13]
		C1 to C10	Enzymatic	1-ethyl-3-methylimidazolium hexafluorophosphate	27.4–32.9% (C8)	[28]
FLV	Naringin	C8, C10, C12	Enzymatic	Acetone *tert*-butanol THF Fatty alcohols	30% (C8), 23% (C10), 24% (C12) 15% (C8), 25% (C10), 16% (C12) 2% (C8/C10), 7% (C12) 8% (C8), 14% (C10), 5% (C12)	[21]
FLV	Rutin	C8, C10, C12	Enzymatic	Acetone *tert*-butanol THF Fatty alcohols	18% (C8), 20% (C10), 21% (C12) 19% (C8), 23% (C10), 20% (C12) 2% (C8/C10), <2% (C12) 10% (C8), 8% (C10), 5% (C12)	[21]
FLV	Malvidin-3- *O*-glucoside	Stearoyl chloride	Chemical	Acetonitrile	Data not available	[42]
FLV	Procyanidin B4	Stearoyl chloride	Chemical	Dimethylformamide, benzyl bromide, potassium carbonate, acetonitrile, triethylamine, dichloromethane, dimethylaminopyridine	70%	[42]
FLV	Delphinidin-3- *O*-sambubioside	Octanoyl chloride	Chemical	Dimethylfomamide	59–95%	[19]
		Octanoic acid	Enzymatic	Fatty acid, 2-methyl-2-butanol	15%	[43]
FLV	Delphinidin-3- *O*-glucoside	Octanoic acid	Enzymatic	Fatty acid, 2-methyl-2-butanol	28%	[43]
FLV	Cyanidin-3- *O*-sambubioside	Octanoyl chloride	Chemical	N,N-dimethylformamide, triethylamine, octanoyl chloride	Data not available	[20]
FLV	(+) Catechin	C12	Enzymatic	Lauric acid. Methanol, ethanol, acetone, isopropanol, tetrahydrofuran, ethyl acetate, termamyl alcohol, pentane, petroleum ether	>70%, >80%, >80%, >60%, >50%, >50%, >40%, <10%, <10%	[44]
FLV	(-) Epicatechin	C12	Enzymatic	Lauric acid. Methanol, ethanol, acetone, isopropanol, tetrahydrofuran, ethyl acetate, termamyl alcohol, pentane, petroleum ether	>70%, >80%, >80%, >60%, >50%, >50%, >40%, <10%, <10%	[44]
FLV	(-) epicatechin-3-*O*-gallate	C12	Enzymatic	Methanol, ethanol, acetone, isopropanol, tetrahydrofuran, ethyl acetate, termamyl alcohol, pentane, petroleum ether	>70%, >80%, >80%, >60%, >50%, >50%, >40%, <10%, <10%	[44]
FLV	(-) epigallocatechin	C12	Enzymatic	Methanol, ethanol, acetone, isopropanol, tetrahydrofuran, ethyl acetate, termamyl alcohol, pentane, petroleum ether	>70%, >80%, >80%, >60%, >50%, >50%, >40%, <10%, <10%	[44]
		C18, EPA and DHA	Chemical	stearoyl, eicosapentaenoyl and docosahexaenoyl chloride, ethyl acetate	Data not available	[45]
		C3,C8, C12, C18 chlorides, DHA	Chemical	C3,C8, C12, C18 and DHA chlorides, ethyl acetate, pyridine	Data not available	[46]
Ph-OH	Tyrosol	C1, C3, C8, C10, C12, C16, C18 and C18:1	Enzymatic	Fatty acids, 2-methyl-2-propanol/*n*-hexane	99.74% (C2), 95.93% (C3), 85.55% (C8), 75.42% (C10), 73.33% (C12), 69.95% (C16), 66.95% (C18), 57% (C18:1)	[47]
		C18:1	Enzymatic	Methyl oleate, *t*-butanol	Data not available	[14]
Ph-OH	Hydroxytyrosol	C8, C16	Enzymatic	Fatty acids	Data not available	[37]
		C8	Enzymatic	Diethyl ether *n*-pentane, *n*-hexane Chloroform, dichloromethane, tetrahydrofuran	85% 70–80% 20%	[26]
STB	Resveratrol	C3,C4,C6, C8,C10,C12, C14, C16, C18,C18:1, EPA and DHA chlorides	Chemical	Fatty acids, ethyl acetate, pyridine	Data not available	[48]
ELT	Punicalagin	Octanoyl/ dodecyl chloride	Chemical	DMF, acetonitrile, triethylamine, chlorides	Data not available	[49]

HBZ-PA, hidroxybenzoic phenolic acids; HCM-PA, hidroxycinnamic phenolic acids; FLV, flavonoids; Ph-OH, phenyl alcohols; STB, stilbens; ELT, ellagitannins.

**Table 2:** Free radical scavenging behavior of phenolipids determined by DPPH assay.

Phenolipid	Polyphenolic Parent Compound	Lipophilic Domain	Results	References
Ferulates	Ferulic acid	C1 to C12	Ferulates ≈ Ferulic acid	[23]
Gallates	Gallic acid	C1, C3, C12 and C18	Gallates > Gallic acid	[23]
Dihydrocaffeate	Dihydrocaffeic acid (DHCA)	C18	DHCA > linolenyc Dihydrocaffeate	[32]
Clorogenates	5- caffeoylquinic acid (5-CQA)	C1, C4, C8, C12, C16, C18 and C20	C4/C8 clorogenates > 5-CQA	[41]
Rosmarinates	Rosmarinic acid (RA)	C4, C8, C12 and C20	C8/C12 rosmarinates > RA	[12]
Caffeates	Caffeic acid (CA)	C1, C2, C3, C4, C6, C8, C10,C12, C14, C16	C1-C8 caffeates > CA> C10-C16 caffeates	[31]
Hydroxytyrosol esters	Hydroxytyrosol (HTy)	C1 to C16	HTy esters ≈ Hydroxytyrosol	[51]
Sinaptic acid ester	Sinaptic acid	C8	Octyl sinaptic acid ester > sinaptic acid	[52]
Ferulate	Ferulic acid	C8	Octyl ferulate > ferulic acid	[52]
Caffeate	Caffeic acid	C8	Octyl caffeate > caffeic acid	[52]
*p*-coumarilate	*p*-coumaric acid (pCA)	C8 and C16	*p*CA > C8 *p*CA > C16 kCA	[37]
Dihydrocaffeates	Dihydrocaffeic acid (DHCA)	C8 and C16	C16 DHCA < C8 DHCA < DHCA	[37]
Caffeates	Caffeic acid (CA)	C8 and C16	C16 CA > C8 CA > CA	[37]
Hydroxytyrosol esters	Hydroxytyrosol (HTy)	C8 and C16	C8 HTy > HTy > C16 HTy	[37]
Tyrosol esters	Tyrosol (Ty)	C8 and C16	Tyrosol > C8/C16 Tyr Esters	[37]
(+) Catechin monoester	(+) Catechin ((+)Cat)	C18	(+) Catechin mono ester > (+)Cat	[42]
Malvidin-3-glucoside mono- /di- ester	Malvidin-3-glucoside (Mv3glu)	C18	Mv3glu di-ester > Mv3glu mono-ester > Mv3glu	[42]
Procyanidin B4 di-ester	Procyanidin B4	C18	Procyanidin B4 > Procyanidin B4 di-ester	[42]
Caffeates	Caffeic acid	C1, C4, C8, C12, C16, C18 and C20	Caffeates ≈ Cafeic acid	[13]
Ferulates	Ferulic acid	C1, C4, C8, C12, C16, C18 and C20	Ferulates < Ferulic acid	[13]
Coumarilates	Coumaric acid (CmA)	C1, C4, C8, C12, C16, C18 and C20	C8 Coumarilate > CmA > (C1,4,12,16,18) Coumarilates	[13]
(+) Catechin mono-ester	(+) Catechin ((+)Cat)	C12	(+) Catechin mono ester > (+)Cat	[44]
Epicatechin mono-ester	(-) Epicatechin ((-) EC)	C12	Epicatechin mono-ester > (-)EC	[44]
Epicatechin-3- *O*-gallate mono- ester	(-) Epicatechin-3- *O*-gallate ((-)EC3OG)	C12	Epicatechin-3- *O*-gallate mono- ester > (-)EC3OG	[44]
Epigallocatechin di-ester	(-) Epigallocatechin ((-)EGC)	C12	Epigallocatechin di-ester > (-)EGC	[44]
Protocatechuates mono-esters	Protocatechuic acid (PA)	C1, C2, C3	Propyl PA > Ethyl PA > Methyl PA > PA	[29]
Epicallocatechin mono-esters	Epigallocatechin (EGC)	C3,C8, C12, C18 chlorides, docosahexanoic acid (DHA)	EGC > C3 EGC > C8 EGC > C12 EGC > DHA EGC	[46]
Punicalaginate mono-esters	Punicalagin (PG)	C8, C12 chlorides	C8 PG > PGC > C12 PG	[49]
Resveratryl mono-esters	Resveratrol (Rvt)	C3:0, C4:0, C6:0, C8:0, C10:0, C12:0, C14:0, C16:0, C18:0, C18:1, C20:5 and C22:6 Chlorides	Rvt> C18:1 Rvt > C18 Rvt > DHA Rvt > C14 Rvt > C16 Rvt > EPA Rvt > C6 Rvt > C12 Rvt - C8 Rvt-C3 Rvt > C4 Rvt > C10 Rvt	[48]

## Data Availability

Not applicable.

## Abbreviations

The following abbreviations are used in this manuscript:

AAPH	2,2′-azobis(2-amidinopropane) dihydrochloride reagent
ABTS	2,2′-azino-bis(3-ethylbenzothiazoline-6-sulfonic acid reagent
DNA	Deoxyribonucleic acid
FRAP	Ferric Reducing Antioxidant Power assay
MTT	3(4,5-dimethylthiazol-2-yl)2,5-diphenyltetrazolium bromide reagent
